# Regulatory role of pro-Th1 and pro-Th2 cytokines in modulating the activity of Th1 and Th2 cells when B cell and macrophages are used as antigen presenting cells

**DOI:** 10.1186/1471-2172-7-17

**Published:** 2006-08-07

**Authors:** Vinod Singh, Javed N Agrewala

**Affiliations:** 1 Institute of Microbial Technology, Sector 39A, Chandigarh-160036, India

## Abstract

**Background:**

Presence of antigen presenting cells, expression of costimulatory molecules, the strength of first signal and cytokine milieu are quite important in influencing the reactivation of differentiated Th1 and Th2 cells.

**Results:**

In the present study, we have analyzed the concerted action of pro-Th1 and pro-Th2 cytokines in the presence of B cells, peritoneal and splenic macrophages as antigen presenting cells and varied concentration of first (anti-CD3 Ab) and second (B7-1 transfectant) signals on the proliferation and cytokine secretion by Th1 and Th2 cells. Interesting observations were made that IFN-γ significantly augmented the secretion of IL-4 by Th2 cells when either B cells or splenic or peritoneal macrophages were used as APC. Further, IFN-γ significantly inhibited the proliferation of Th1 cells only in the presence of peritoneal macrophages. We have also observed that B cells could significantly respond to cytokines to further enhance the proliferation and cytokine release by Th1 and Th2 cells. But not much effect on addition of exogenous cytokines IL-1, IL-4, IL-5, IL-12 was observed on the proliferation of Th1 and Th2 cells in the presence of macrophages. In contrast, both IFN-γ and IL-2 significantly enhanced the production of IL-4 and IL-5 respectively, by Th2 cells in presence of B cells, splenic and peritoneal macrophages. Another important observation was that the addition of B7-1 transfectants in the cultures, which were stimulated with low dose of anti-CD3 Ab significantly, enhanced the proliferation and cytokine secretion.

**Conclusion:**

This study indicates involvement of different type of APCs, cytokine milieu, dose of first and second signals in a concerted manner in the outcome of the immune response. The significance of this study is that the immunization with antigen along with costimulatory molecules may significantly reduce the dose of antigen and can generate better immune response than antigen alone.

## Background

On the basis of lymphokines profile, CD4^+ ^T cells can be differentiated into Th1 and Th2 subtypes. Th1 cells secrete IL-2, IFN-γ, lymphotoxin, etc., and are mainly involved in the generation of cell-mediated immunity. Th2 cells secrete IL-4, IL-5, IL-6, etc., and are generally involved in humoral immunity [1,2]. These differentiated cells are the effector arms of the immune system that respond more rapidly and effectively to pathogens that have been encountered previously and reflect the pre-existence of clonally expanded populations of antigen-specific lymphocytes. Hence it is quite crucial to study the factors that govern the reactivation of the differentiated Th1 and Th2 cells. Many factors such as antigen presenting cells (APCs), pro-Th1 and proTh-2 cytokines, strength of first signal and expression of costimulatory molecules may influence the activation of Th1 and Th2 cells [3-6]. Antigen presenting cells like B cells, macrophages and dendritic cells are critical players in the immune response. Dendritic cells are potent activators of naïve T cells but it has been reported that B cells are the most potent APCs in inducing proliferation of differentiated Th2 cells and splenic macrophages for differentiated Th1 cells [7]. Further, it has also been suggested that peritoneal macrophages are favored APCs for both Th1 and Th2 cells. However, it has also been shown that B cells can function, as APCs for Th1 cells but for Th2 cells, the presence of IL-1 is required [8]. The strength of signaling through MHC-peptide (MHCP) can also affect the activity of terminally differentiated Th1 and Th2 cells. It has been reported that differential signaling via anti-CD3 mAb inhibits IL-2-dependent proliferation of Th1 but not Th2 cells [9,10]. The expression of costimulatory molecules on APCs is also crucial for the optimum activation of T cells and therefore may have relevance with diseases [11]. Array of costimulatory molecules are expressed on the surface of APCs but the best-defined costimulators are B7-1 and B7-2 [12-14]. It has been reported that B7-1 and B7-2 and presence of pro-Th1 (IL-2, IL-12, IFN-γ) and pro-Th2 (IL-4) cytokines can differentially regulate the activation of Th1 and Th2 cells [14-16].

It appears that APCs, costimulatory molecules and pro-Th1 and pro-Th2 cytokines operate in a complex and concerted manner that can manipulate the activation of effector Th1 and Th2 cells. To address this issue, we studied the role of pro-Th1 and pro-Th2 cytokines in the activation of Th1 and Th2 cells when B cells, splenic and peritoneal macrophages were used as APCs. Further, the role of different doses of first (anti-CD3 Ab) and second signals (B7-1 transfectant) was also studied. It was observed that regulation of the activation of Th1 and Th2 cells is not a mere function of cytokines but can be significantly modulated by the type of APCs and dose of first and second signals.

## Results

**Figure 1:**
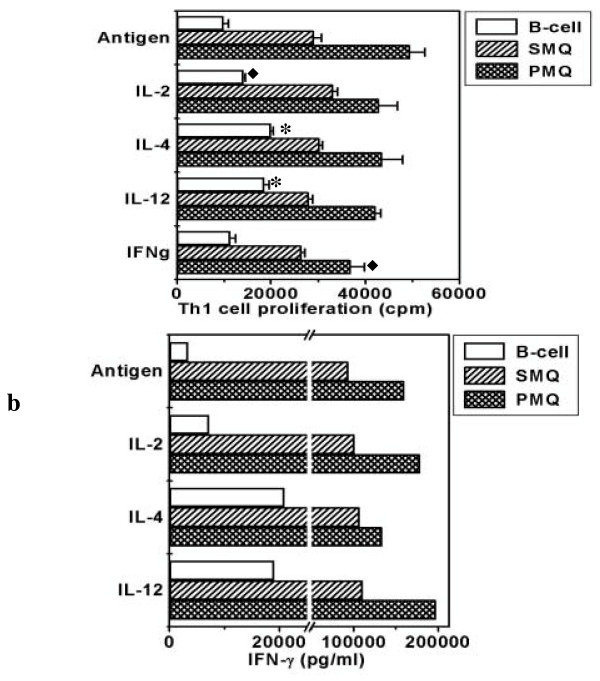
Influence of Pro-Th1 (IL-2, IL-12, IFN-γ) and Pro-Th2 (IL-4) cytokines on the proliferation and IFN-γ secretion when B cells, splenic macrophages and peritoneal macrophages were used as APCs. pGL-10 Th1 cells were cultured with B cells, splenic and peritoneal macrophages and ovalbumin (200 μg/ml). Cytokines IL-2, IL-4, IL-12 and IFN-γ were also added in the cultures. For proliferation, the cultures were pulsed after 48 h with ^3^H-thymidine (0.5 μCi/well) and harvested after last 16 h of incubation period. Data are expressed as mean ± SEM of triplicate wells (Fig. 1a). For IFN-γ, the supernatants were collected from the triplicate wells after 48 h of the initiation of cultures, pooled and estimated by ELISA. (Fig. 1b). The control cultures comprising of Th1 cells, Th1 cell+Ag, APCs+Ag, APCs+Th1 cells showed no discernible change. The data presented are from three independent experiments. '*' Represents p < 0.001 and '◆' represents p < 0.05. SMQ and PMQ represent splenic and peritoneal macrophages respectively.

Influence of pro-Th1 (IL-2, IL-12 and IFN-γ) and pro-Th2 (IL-4) cytokines on the proliferation and IFN-γ secretion by Th1 cells when B cells, splenic and peritoneal macrophages were used as a source of APCs. In the present study we have chosen very well characterized and widely studied Th1 (pGL-10, AE7) and Th2 clones (D10G4.1). Antigen-pulsed and gamma-irradiated B cells and macrophages were used as APCs. We observed the maximum proliferation of Th1 cells with peritoneal macrophages (49590 ± 3056 cpm) followed by splenic macrophages (29057 ± 1639 cpm) (Fig. 1a). Minimum proliferation (9945 ± 1020 cpm) was observed when B cells were used as a source of APCs. Interestingly, when either IL-4 or IL-12 was added into the cultures, there was a significant (p < 0.001) enhancement in the proliferation of Th1 cells when B cells were used as a source of APCs. It may also be noted here that in all the experiments, most potent dose of cytokines was used that was selected after conducting dose dependent experiments (data not shown). There was a marginal increase with IL-2 (p < 0.05) but IFN-γ failed to bring any noticeable change in the proliferation of Th1 cells when B cells were used as a source of APCs. Surprisingly, addition of cytokines (IL-2, IL-4, IL-12, IFN-γ) could not induce any change in the proliferation with splenic macrophages. In contrast, IFN-γ (p < 0.05) inhibited the proliferation when peritoneal macrophages were used (Fig. 1a).

We observed maximum secretion of IFN-γ by Th1 cells when peritoneal macrophages (159700 pg/ml) were used as a source of APCs followed by splenic macrophages (93530 pg/ml) and B cells (3466 pg/ml). Interestingly, the presence of IL-4 (505%) and IL-12 (450%) significantly enhanced the secretion of IFN-γ when B cells, but not splenic and peritoneal macrophages were employed to present antigen (Fig. 1b). The presence of IL-2 could not modulate any change in IFN-γ release in the presence of either B cells or macrophages.

**Figure 2:**
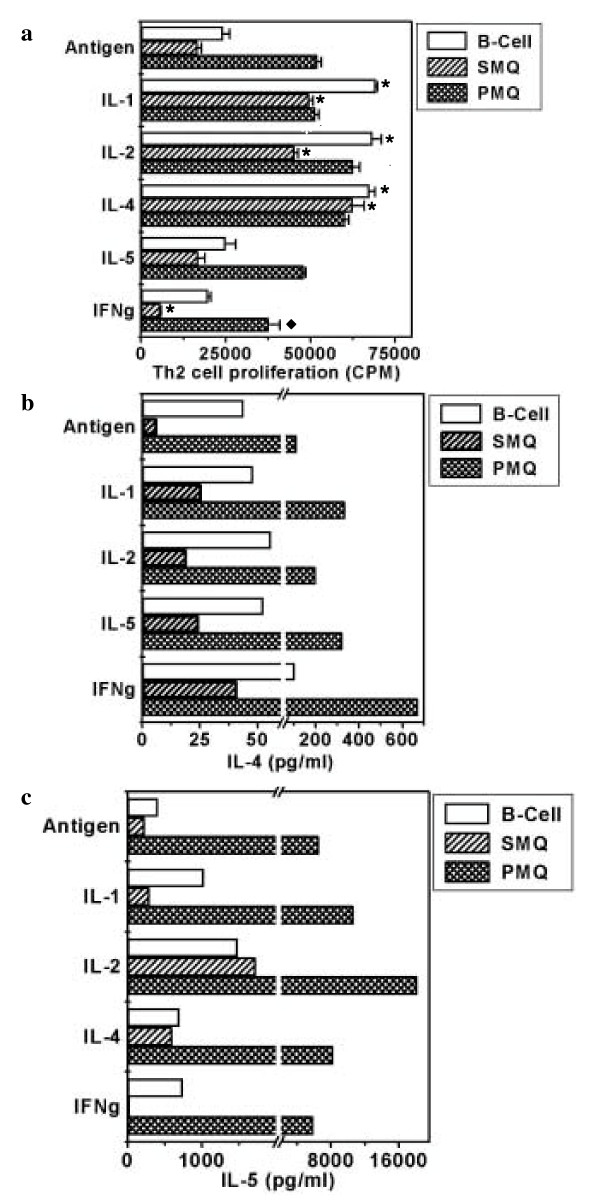
Influence of Pro-Th1 (IL-2, IFN-γ) and Pro-Th2 (IL-1, IL-4, IL-5) cytokines on the proliferation and IL-4 and IL-5 secretion by Th2 cells when B cells, splenic macrophages and peritoneal macrophages were used as APC. D10G4.1 Th2 cells were cultured with B cells, splenic and peritoneal macrophages and conalbumin (100 μg/ml). Cytokines IL-1, IL-2, IL-4 or IL-5 and IFN-γ were also added in the cultures. Cultures with peritoneal macrophages also received 1 mM aminoguanidine. For proliferation, the cultures were pulsed after 48 h with ^3^H-thymidine (0.5 μCi/well) and harvested after last 16 h of incubation period. Data are expressed as mean ± SEM of triplicate wells (Fig. 2a). For IL-4 (Fig. 2b) and IL-5 (Fig. 2c), the supernatants were collected from the triplicate wells after 48 h of the initiation of cultures, pooled and estimated by ELISA. The data are expressed as pg/ml. The control cultures comprising Th2 cells, Th2 cell+Ag, APCs+Ag, APCs+Th2 cells showed no apparent change. The data presented are from three independent experiments. '*' Represents p < 0.001 and '◆' represents p < 0.05. SMQ and PMQ represent splenic and peritoneal macrophages respectively.

Influence of pro-Th1 (IL-2 and IFN-γ) and pro-Th2 (IL-1, IL-4 and IL-5) cytokines on the proliferation and IL-4 and IL-5 production by Th2 cells when B cells, splenic and peritoneal macrophages were used as a source of APCs. Peritoneal macrophages (51978 ± 1370 cpm) were the most potent APCs followed by B cells (24326 ± 1871 cpm) in inducing the proliferation of Th2 cells. Splenic macrophages (16734 ± 1085 cpm) induced lowest proliferation. Interestingly, IL-1, IL-2 and IL-4 significantly (p < 0.001) enhanced the proliferation of Th2 cells when B cells and splenic macrophages were used as a source of APCs (Fig. 2a). No change was observed with IL-5. However, the proliferation was significantly inhibited by IFN-γ in the presence of splenic (p < 0.001) and peritoneal (p < 0.05) macrophages.

Th2 cells maximally secreted IL-4 when peritoneal macrophages (112.5 pg/ml) were used as a source of APCs followed by B cells (44 pg/ml) (Fig. 2b). Splenic macrophages (6.47 pg/ml) were poor stimulator of Th2 cells for IL-4 secretion. Interestingly, addition of IFN-γ significantly augmented the yield of IL-4 irrespective of the type of APCs used in the study. Addition of IL-1, IL-2 and IL-5 significantly enhanced the secretion of IL-4 when splenic and peritoneal macrophages but not B cells were used as APCs.

There was dramatic increase in the release of IL-5 by Th2 cells with peritoneal macrophages (6533 pg/ml). B cells (420 pg/ml) and splenic macrophages (230 pg/ml) could also induce moderate production of IL-5. Addition of IL-2 significantly increased the production of IL-5 with all the three APCs (Fig. 2c). Further, incorporation of IL-1 into B cell cultures also enhanced the secretion of IL-5. Addition of IL-4 and IFN-γ in the cultures failed to show any dramatic change in the secretion of IL-5.

**Figure 3:**
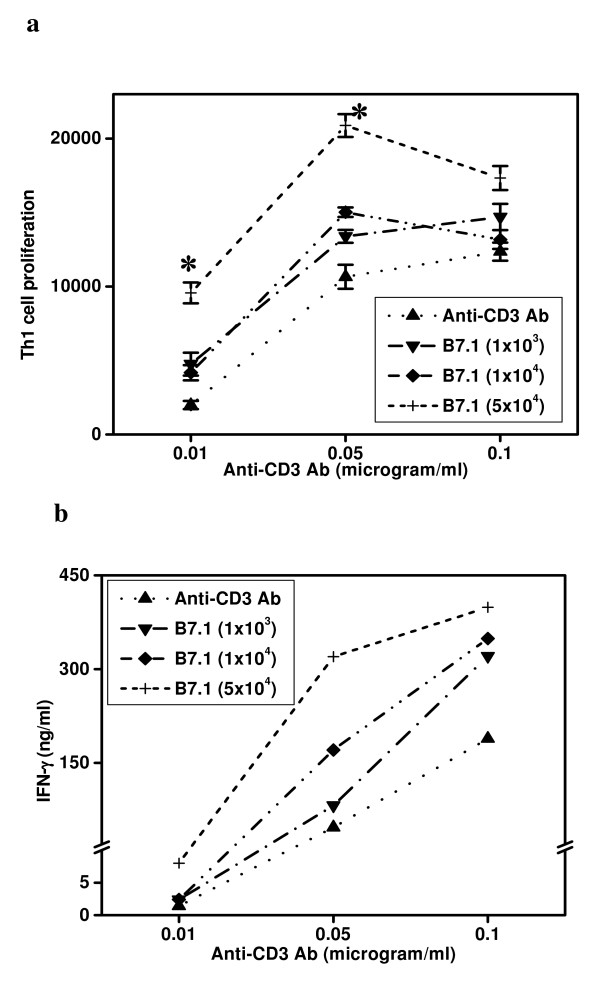
Influence of the potency of first and second signals in the proliferation and IFN-γ secretion by Th1 cells. Cultures were set with pGL-10 Th1 cells (5 × 10^4^/well) and the indicated concentrations of plate-bound anti-CD3 mAb (0.01 μg/ml) and in the presence of different concentrations of CHO-B7-1 (1 × 10^3^/well, 1 × 10^4^/well, 5 × 10^4^/well). The cultures were pulsed after 48 h with ^3^H-thymidine (0.5 μCi/well) and harvested after last 16 h of incubation period. Data are expressed as mean ± SEM of triplicate wells (Fig. 3a). Production of IFN-γ (Fig. 3b) was estimated by ELISA in the pooled culture supernatants collected from the triplicate wells after 48 h of the initiation of cultures. The data is expressed as ng/ml. The control cultures comprising Th1 cells, Th1 cells+CHO-B7-1, Th1 cells+CHO cells, CHO-B7-1 cells+anti-CD3 Ab, CHO-B7-1 cells showed no noticeable change. The data presented are from three independent experiments. '*' Represents p < 0.001.

Influence of first and second signals in the proliferation and IFN-γ secretion by Th1 cells. After studying the role of APCs, we also monitored the role of different doses of first (anti-CD3 Ab) and second (B7-1 transfectants) signals in the activation of Th1 and Th2 cells (Fig. 3a). The first signal was provided using low (0.01 μg/ml), moderate (0.05 μg/ml) and high concentration (0.1 μg/ml) of anti-CD3 Ab and second signal in the form of low (5 × 10^3 ^cells/well), moderate (1 × 10^4 ^cells/well) and high number (5 × 10^4 ^cells/well) of B7-1 transfectants. Moderate to high but not low concentration of anti-CD3 Ab induced optimum proliferation of Th1 cells (Fig. 3a). Interestingly, addition of high concentration of B7-1 transfectants (5 × 10^4 ^cells/well) into the cultures also successfully enhanced the proliferation of the cells stimulated with low strength of first signal (p < 0.001) (Fig. 3a). Even though, moderate dose of anti-CD3 Ab induced the optimum level of proliferation but the addition of high dose of B7-1 transfectants further enhanced the proliferation of Th1 cells (p < 0.001). In contrary, delivery of strong first signal in association with high number of B7-1 transfectants showed inhibitory effect. Low dose of first signal alone failed to induce the secretion of IFN-γ (Fig 3b). However, with the increase in the concentration of anti-CD3 Ab (0.05–0.1 μg/ml), detectable level of IFN-γ was noticed. When T cells were stimulated with low dose of first signal, B7-1 costimulation showed marginal increase in the IFN-γ yield. Interestingly, addition of low to high dose of B7-1 transfectants in the Th1 cell cultures stimulated with either moderate or strong first signals could substantially enhance the secretion of IFN-γ (Fig 3b). Similar results were also obtained in the case of AE7 Th1 cells (data not shown).

**Figure 4:**
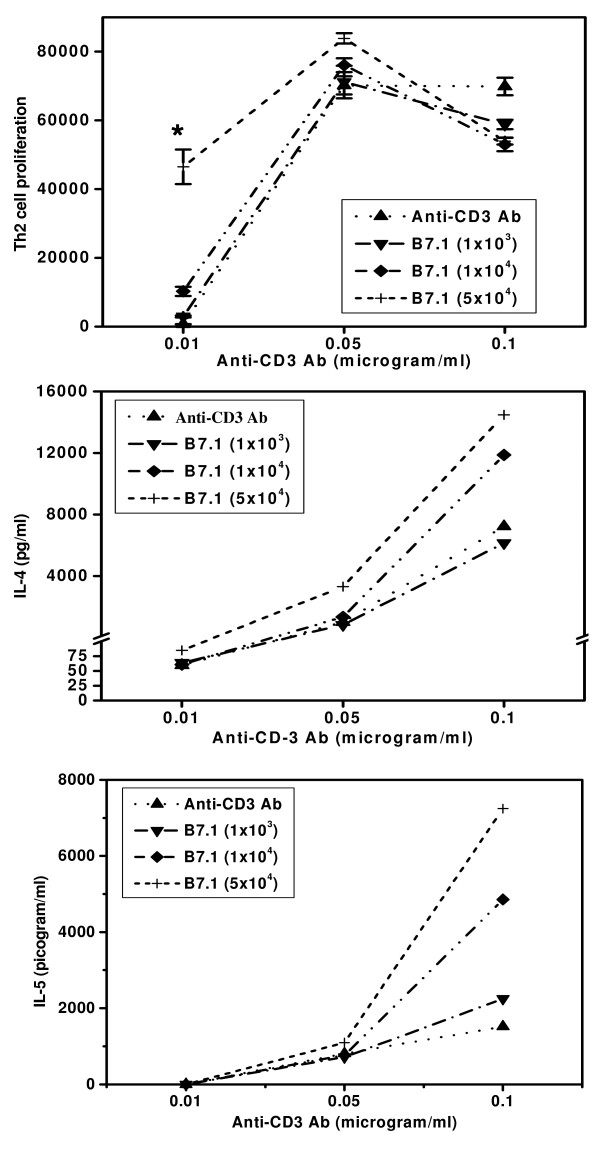
Influence of the potency of first and second signals on the proliferation of Th2 cells and secretion of IL-4 and IL-5. Cultures were set with (5 × 10^4^/well) D10G4.1 Th2 cells and the indicated concentrations of plate-bound anti-CD3 mAb (0.01 μg/ml) and in the presence of different concentrations of CHO-B7-1 (1 × 10^3^/well, 1 × 10^4^/well, 5 × 10^4^/well). For proliferation (Fig. 4a), all the cultures were pulsed after 48 h with ^3^H-thymidine (0.5 μCi/well) and harvested after the last 16 h of incubation period. Data are expressed as mean ± SEM of triplicate well. Production of IL-4 (Fig. 4b) and IL-5 (Fig. 4c) was estimated by ELISA. The supernatants were pooled from the triplicate wells after 48 h of the initiation of cultures. The data are expressed as pg/ml. The control cultures comprising of Th2 cells, Th2 cells+CHO-B7-1, Th2 cells+CHO cells, CHO-B7-1 cells+anti-CD3 Ab, CHO-B7-1 cells showed no remarkable change. The data presented are from four independent experiments. '*' Represents p < 0.001.

Importance of the first and second signals in proliferation and cytokine (IL-4 and IL-5) secretion by Th2 cells. It was observed that moderate dose of anti-CD3 Ab induced the proliferation of Th2 cells (D10G4.1) (Fig 4a). Like Th1 cells, addition of high dose of B7-1 transfectants augmented the proliferation of Th2 cells stimulated with low dose of anti-CD3 Ab (p < 0.001). Interestingly, B7-1 signal showed marginal increase in the proliferation with the moderate dose but inhibition was observed with high dose of first signal (Fig 4a). Also delivery of B7-1-mediated signal could not show any change in the secretion of IL-4 when cells were stimulated with low dose of first signal (Fig 4b). However, high dose of first signal alone induced significant level of IL-4 secretion. Unlike proliferation, addition of moderate to high, but not low numbers of B7-1 transfectants further enhanced the secretion of IL-4 by Th2 cells that were stimulated with high dose of anti-CD3 Ab. As observed in the case of IL-4 secretion, similar results were demonstrated in the case of IL-5 production (Fig 4c).

**Figure 5:**
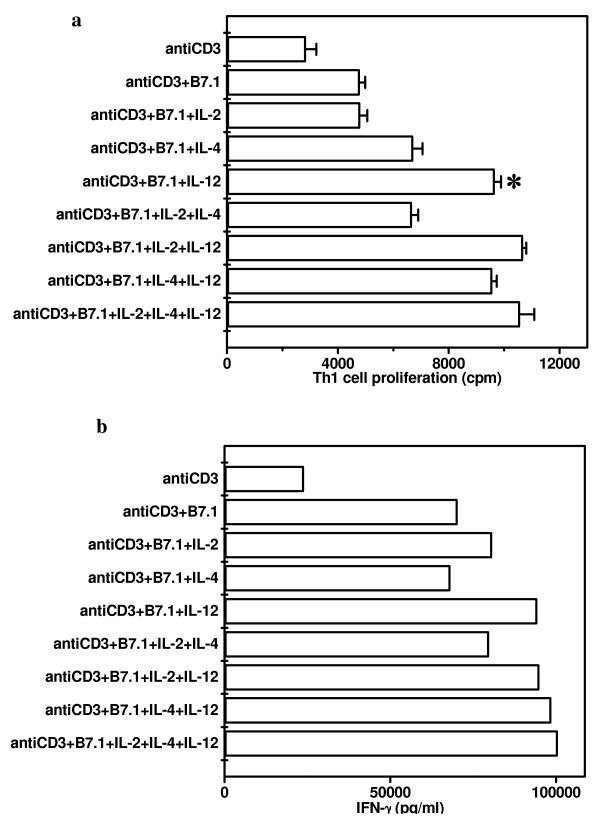
Influence of Pro-Th1 (IL-2 and IL-12) and Pro-Th2 (IL-4) cytokines on the proliferation and IFN-γ secretion by Th1 cells stimulated with anti-CD3 Ab and B7-1. pGL-10 Th1 cells were cultured with anti-CD3 Ab (0.01 μg/ml) in the presence of CHO-B7-1 (5 × 10^4^/well). Cytokines IL-2, IL-4 or IL-12 alone or in combinations were also added into the cultures. For proliferation (Fig. 5a), cultures were pulsed after 48 h with ^3^H-thymidine (0.5 μCi/well) and were harvested after last 16 h of incubation period. Data are expressed as mean ± SEM of triplicate wells. IFN-γ (Fig. 5b) was estimated by ELISA in the pooled culture supernatants collected from the triplicate wells after 48 h of the initiation of cultures. The control cultures comprising of Th1 cells, CHO-B7-1+Th1 cells, CHO+Th1 cells, CHO-B7-1, CHO showed no noticeable change. The data presented are from three independent experiments. '*' Represents p < 0.001.

Influence of pro-Th1 (IL-2 and IL-12) and pro-Th2 (IL-4) cytokines in proliferation and IFN-γ secretion by Th1 cells stimulated with anti-CD3 Ab and B7-1. It was observed that IL-12 significantly (p < 0.001) enhanced the proliferation of Th1 cells that were activated with anti-CD3 Ab and B7-1 (Fig 5a). We also observed that IL-12 enhanced the secretion of IFN-γ by Th1 cells stimulated with anti-CD3 Ab and B7-1 (Fig 5b). In control cultures, anti-CD3 Ab alone induced the secretion of IFN-γ and addition of B7-1 transfectants substantially enhanced its production.

**Figure 6:**
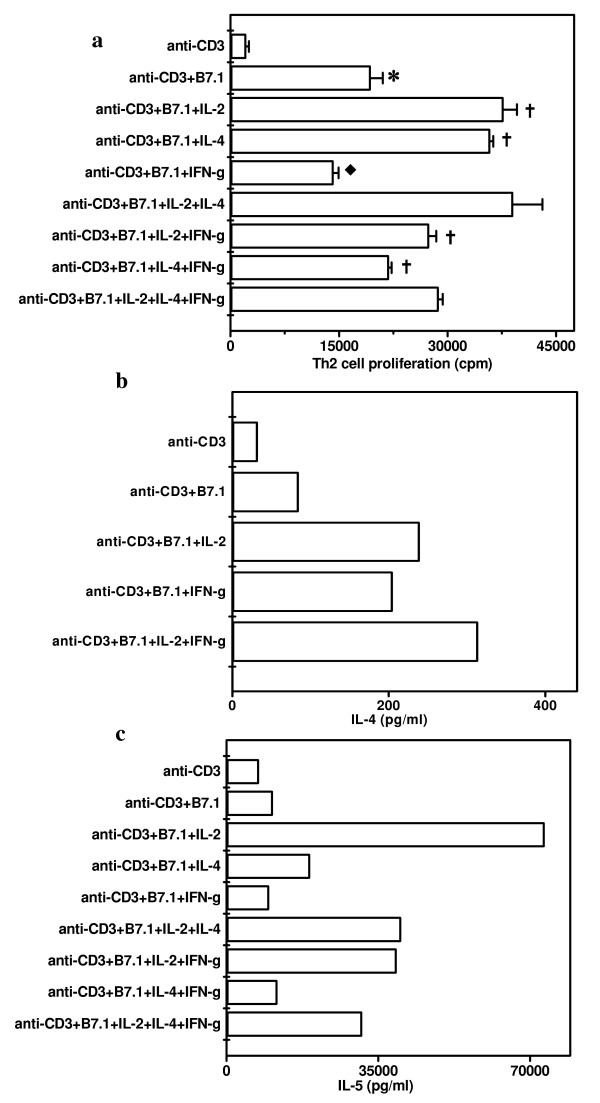
Influence of Pro-Th1 (IL-2 and IFN-γ) and Pro-Th2 (IL-4) cytokines on the proliferation and IL-4 and IL-5 secretion by Th2 cells stimulated with anti-CD3 Ab and B7-1. D10G4.1 Th2 cells were cultured with anti-CD3 Ab (0.01 μg/ml) in the presence of CHO-B7-1 (5 × 10^4^/well). Cytokines IL-2, IL-4 or IFN-γ alone or in combinations were also added in to the cultures. For the proliferation (Fig. 6a), cultures were pulsed after 48 h with ^3^H-thymidine (0.5 μCi/well) and were harvested after last 16 h of incubation period. Data are expressed as mean ± SEM of triplicate wells. IL-4 (Fig. 6b) and IL-5 (Fig. 6c) were estimated by ELISA in the pooled supernatants collected from the triplicate wells after 48 h of the initiation of the cultures. The control cultures comprising Th2 cells, CHO-B7-1+Th2 cells, CHO+Th2 cells, CHO-B7-1, CHO showed no considerable change except that IL-5 secretion was 1995 pg/ml when D10G4.1 Th2 cells were incubated with CHO-B7.1 in the absence of anti-CD3 Ab. The data presented are from three independent experiments. '*' Represents p < 0.001, '†' represents p < 0.01 and '◆ ' represents p < 0.05.

Influence of pro-Th1 (IL-2 and IFN-γ) and pro-Th2 (IL-4) cytokines in proliferation and IL-4 and IL-5 secretion by Th2 cells stimulated with anti-CD3 Ab and B7-1. It was observed that both IL-2 and IL-4 significantly (p < 0.01) enhanced the proliferation of Th2 cells activated with anti-CD3 Ab and B7-1 (Fig 6a). Interestingly, IFN-γ not only inhibited (p < 0.05) the proliferation of Th2 cells activated with anti-CD3 Ab and B7-1 but also decreased IL-4 and IL-2 mediated proliferation significantly (p < 0.01). In the control wells, anti-CD3 Ab alone showed marginal proliferation but when B7-1 transfectants were added, significant proliferation (p < 0.001) was noticed. IL-2 further augmented the secretion of IL-4 when Th2 cells were stimulated with anti-CD3 Ab and B7-1 transfectants (Fig 6b). Surprisingly, like IL-2, IFN-γ also upregulated the secretion of IL-4. When IL-2 and IFN-γ were used in combination, they showed additive effect. IL-2 induced remarkable amount of IL-5 production by Th2 cells that were activated with anti-CD3 Ab and B7-1 (Fig 6c). We also observed that IL-5 secretion augmented by IL-2 was significantly decreased on addition of IL-4 and IFN-γ into the cultures. In the control cultures, we detected significant secretion of IL-5 by Th2 cells activated with anti-CD3 Ab but there was no major change with the addition of B7-1 transfectants.

## Discussion

Both Th1 and Th2 cells play a major role in generating effective immune response. But Th1 and Th2 cells can also be devastating in some disease conditions like allergy and autoimmunity respectively [7,18,19]. Many factors like the strength of first signal, expression of costimulatory molecules, presence of cytokines, interaction with different APCs, etc., can influence the activation of Th1 and Th2 cells [7-10,13-16]. But how the concerted action of these factors influence their activation is not very well documented. It is therefore of enormous interest to know how these factors govern the proliferation, cytokine production and development of effector function of Th1 and Th2 cells. We therefore conducted this study to investigate the role of pro-Th1 and pro-Th2 cytokines in the activation of rested Th1 and Th2 cells. This activation is caused by different concentration of anti-CD3 Ab and B7-1 transfectants as a source of first signal and second signals respectively and B cells, splenic and peritoneal macrophages as APCs.

We observed that as compared to B cells and splenic macrophages, maximum proliferation and cytokine secretion by Th1 and Th2 cells was observed when peritoneal macrophages were used as APCs. This may be due to the fact that thioglycollate elicited peritoneal macrophages release cytokines and express high level of costimulatory molecules [19,20]. Interestingly, IFN-γ, in the presence of peritoneal macrophages but not splenic macrophages and B cells suppressed the proliferation of Th1 cells mainly. The reason for this observation may be explained by the fact that on interaction with Th1 cells, macrophages produce nitric oxide (NO). Thioglycollate elicited macrophages are high producers of IL-12. IL-12 skews immune response towards Th1 cells, which in turn produce IFN-γ. Recently, it has been reported in the case of T. crassiceps that peritoneal macrophages impair T cell proliferation by secreting NO. Nitric oxide also contributes to asthma pathogenesis by selective down-regulation of Th1 responses [21-23]. It may be inferred from this finding that activated macrophages interact with Th1 cells in a manner that leads to high production of IFN-γ that can inhibit their proliferation and impair their capacity to respond to cytokines. This strategy may be utilized by intracellular pathogens in taming macrophages in their favor and thereby suppressing Th1 response [21-25]. Therefore targeting antigen to other APCs like B cell and dendritic cell for the activation of Th1 cells during intracellular infection of macrophages may avoid immunosuppression. Our observation shows that B cells can significantly respond to cytokines to further enhance the proliferation and cytokine release by Th1 and Th2 cells (Fig. 1, 2). Addition of exogenous cytokines (IL-1, IL-4, IL-5, IL-12) had no effect on the proliferation of Th1 and Th2 cells in the presence of macrophages (Fig. 1, 2). In contrast, IFN-γ and IL-2 significantly enhanced the production of IL-4 and IL-5 respectively by Th2 cells in presence of B cells, splenic and peritoneal macrophages. This indicates that signals delivered by IFN-γ or IL-2 worked irrespective of the type of APCs. IL-5 is an important mediator of eosinophilia in mice with parasite infections. It has also been shown that mice administered IL-2 developed eosinophilia. Therefore our study indicates that precaution should be taken while using IL-2 for immunomodulation, since despite inhibiting, it induces eosinophilia [26]. Further, the finding that IFN-γ induces IL-4 secretion by Th2 cells is in contrast with the earlier findings but recently, it has been reported that IFN-γ has dual role in the activation of Th2 cells [27,28]. High concentration of IFN-γ can inhibit whereas low dose can prime cells to develop into IL-4 secreting Th2 cells. This indicates that depending on the concentration, a cytokine can have dual role on Th1 and Th2 cells and subsequently on the outcome of the disease. Further, these observations indicate that cytokine-mediated regulation of polarization of CD4^+ ^T cells to the Th1 or Th2 phenotype is more complex than originally envisioned in the studies that examined the dominant role of cytokines [27-29].

Our observation also indicates that IFN-γ can act directly on differentiated Th2 cells to enhance IL-4 production and not necessarily through APCs. IFN-γ enhanced the secretion of IL-4 when Th2 cells were stimulated with anti-CD3 Ab and B7-1 transfectant. However, this does not rule out the possibility of IFN-γ working on Th2 cells through APCs as well. This is evident by the fact that the production of IL-4 was higher when stimulated with APCs as compared to anti-CD3 Ab and B7-1 transfectants (Fig. 2b, 6b).

Another important observation of the study was that appropriate dose of first signal and expression of costimulatory molecules is quite crucial for the optimum proliferation and cytokine secretion by Th1 and Th2 cells. Interestingly, addition of B7-1 transfectants in the cultures using low dose of anti-CD3 Ab (0.01–0.05 μg/ml), significantly enhanced the proliferation and cytokine secretion. The significance of this observation is that it may have important application in vaccine design and development. Formulating a device using costimulatory molecules may elicit optimal T-cell response with even low dose of antigen. Therefore, one can overcome the problems related to high dose inoculation of antigen. We also observed that a high dose of first signal delivered in the form of anti-CD3 Ab inhibits the proliferation of Th1 and Th2 cells. This indicates that high dose of antigen and constant stimulation can suppress the T cells. This may explain why the immune response generated due to DNA vaccination fails to evoke a better response than protein antigens. Further, constant stimulation with high dose of antigen can limit protective function of T cells [29-32].

Thus it is quite obvious from our study that not only the presence of particular cytokines, but also the type of APCs and the dose of first and second signals may have significant impact on the activation of Th1 and Th2 cells. So it is quite necessary to take into account the concerted action of the type of APCs, effectiveness of first and second signals and the presence of cytokines while studying the activation of rested Th1 and Th2 cells. This study may be of paramount importance when studying the in vivo immune response generated by the vaccines. Different individuals respond in a different way to vaccines. One possible reason may be the difference in type of APCs that encounter the antigen, the density of antigen, the expression of costimulatory molecules and cytokine milieu. Thus our experiments emphasize the need for detailed analysis of the host immune system before immunizing with a vaccine or immunotherapeutic agents to generate effective immune response. Further, our study suggests that immunization with antigen along with costimulatory molecules may significantly reduce the dose of antigen and can generate better immune response than observed with high dose of antigen.

## Conclusion

Our study demonstrates the involvement of different type of APCs, cytokine milieu, dose of first and second signals in a concerted manner in the outcome of the immune response. The significance of this study is that the immunization with antigen along with costimulatory molecules may considerably decrease the quantity of antigen and can produce better immune response than antigen alone.

## Methods

### Animals

Inbred female BALB/c and C3He mice, 6–10 weeks old, were obtained from the Institute's Animal House Facility, National Institute of Immunology, New Delhi and National Institute of Nutrition, Hyderabad. The animals were fed on standard pellet diet and water ad libitum. All the mice were housed under standard conditions at the Institute's Animal Facility.

### Chemical and reagents

Fetal calf serum was purchased from Harlan Sera Lab (Crawley Down, GB), RPMI 1640, DMEM and HBSS from GIBCO (Grand Island, NY) and L-glutamine, L-pyruvate, penicillin and streptomycin were from Serva (Heidelberg, Germany). Recombinant IL-1 cytokine was procured from Genzyme (Cambridge MA) and recombinant IL-2, IL-4, IL-5, IL-12, and IFN-γ from Pharmingen (San Diego, CA). For ELISA of IL-4, IFN-γ and IL-5, the capture and detection (biotinylated) antibodies were obtained from Pharmingen (San Diego, CA). General chemicals used in the study were procured from the Sigma Chemical Co. (St. Louis, MO). ^3^H-thymidine was the product of Amersham Pharmacia Biotech AB (Uppsala, Sweden).

### Cell lines and hybridomas

The Th2 clone D10G4.1 (TIB-224) was procured from American Type Culture Collection (ATCC), (Rockville, MD). Th1 clone (pGL-10) was a kind gift by Dr. V. M. Sanders, Department of Molecular Virology, Immunology and Medical Genetics, Ohio State University, Columbus, Ohio and Th1 clone (AE7) was a kind gift by Dr. J. Rengarajan, Department of Immunology and Infectious Disease, Harvard School of Public Health, Boston, MA. Anti-CD3 Ab (145.2C11) hybridoma was kind gift from Prof. C. A. Janeway, Jr. (Yale University, New Haven, CT). The CHO-B7-1 transfectants were a kind gift from Dr. A. Ochi, John P. Robarts Research Institute, Ontario, Canada.

### Medium

Cells were cultured in RPMI 1640 or DMEM medium supplemented with 10% FCS, L-glutamine (2 mM), penicillin (50 μg/ml), streptomycin (50 μg/ml), HEPES (100 mM) and 2-ME (0.05 mM).

### Maintenance of antigen-specific Th1 and Th2 cells

The Th1 clones were maintained following the studied protocols [23] and Th2 clones were maintained according to ATCC protocol.

The cultures for Th cell proliferation were set using different concentrations (1 × 10^3^–10^5 ^cells/well) of stimulator cells (macrophages untreated or treated with IFN-γ and LPS-activated B cells) and T cells (2 × 10^4^/well) in the presence or absence of B7-1 Abs. The cultures were also set using HamIg as controls for anti-B7-1 Ab. On day 5 of MLR cultures, [^3^H]-thymidine was added and the cultures were harvested after 16 h and assayed for DNA synthesis by beta scintillation counting.

### Preparation of antigen presenting cells (B cells, splenic and peritoneal macrophages)

A single cell suspension of mice spleens was prepared in a balanced salt solution (BSS). The RBCs were depleted by treatment with ACK lysis solution. The adherent splenic macrophages were removed by plating on the plastic Petri plates (Grenier, Germany) for two hour at 37°C and 7% CO_2_. The adherent cells were washed several times with BSS and then removed by gentle scraping with the rubber policeman from the plate and used as adherent splenic macrophages. The nonadherent cells were treated sequentially on ice for 45 min, with a mixture of anti-Mac-2, Mac-3, Thy1, CD4 and CD8 Abs followed by incubation with baby rabbit complement at 37°C for 45 min. The purity of cells stained with anti-IgM Ab by indirect immunofluorescence, were nearly 80%, as analyzed by flow cytometry (Becton Dickinson, Mountain view, CA).

The PECs (peritoneal exudate cells) were harvested from mice inoculated 4 days previously with 2 to 3 ml of Thioglycollate. The cells were washed with cold BSS. The macrophages were obtained by adhering on plastic Petri's dishes for 1 h at 37°C, followed by washing several times in cold BSS. The cells were then removed from Petri dishes by gentle scraping with the rubber policeman, washed with BSS and resuspended in medium.

### The proliferation and cytokine secretion by Th1 and Th2 cells using different doses of first and second signals

T helper clones (pGL-10 and D10G4.1) were isolated after 7–9 days of activation with antigen-pulsed splenocytes. The dead cells were removed by ficoll-histopaque and washed twice with BSS. The cells (5 × 10^4^/well) were added in 96w flat bottom microtitre plate, pre-coated with different doses of anti-CD3 Ab (0.01–0.1 μg/ml) for 2–3 h in PBS (pH 7.0) at 37°C. The wells were washed three times with BSS. Different concentrations of paraformaldehyde (0.5%) fixed transfectants (5 × 10^3^–5 × 10^4^/well) were then added to T helper cells.

Similar cultures were also set in combination with different cytokines [IL-2 (50 U/ml), IL-4 (100 U/ml), IL-12 (100 U/ml)] for Th1 cells and [IL-2 (50 U/ml), IL-4 (100 U/ml), IFN-γ (100 U/ml)] for Th2 cells. Both Th1 and Th2 cells were also stimulated with anti-CD3 Ab (0.01 μg/ml) and paraformaldehyde fixed transfectants (5 × 10^4^/well). The cells were incubated at 37°C/7% CO_2_. After 48 h, the cultures were pulsed with 0.5 μCi of ^3^H-thymidine and harvested 16 h later by an automatic cell harvester. Radioactivity incorporated was measured by liquid scintillation counting and data were expressed as mean counts per minute (cpm). For the estimation of cytokines (IL-4, IL-5 and IFN-γ), 100 μl of culture supernatant was removed from each set of triplicate wells after 48 h and pooled for ELISA.

### Estimation of cytokines

The cytokines were estimated as per manufacturer instructions (Pharmingen) and expressed as pg/ml using recombinant cytokines as a standard (Pharmingen).

### Proliferation and cytokine secretion by Th1 and Th2 cells in response to APCs (B cells, splenic and peritoneal macrophages) and cytokines (IL-1, IL-2, IL-4, IL-5, IL-12 and IFN-γ)

Th1 cells (5 × 10^4^/well) were cultured with gamma-irradiated B cells, splenic macrophages (1 × 10^5^/well) and peritoneal macrophages (5 × 10^4^/well) as antigen presenting cells in 96 w flat-bottom plate. B cells were irradiated at 500 R and splenic and peritoneal macrophages at 3000 R. Those doses of APCs were chosen which induced the optimum proliferation of Th1 and Th2 cells. In the cultures, IL-2 (50 U/ml), IL-4 (100 U/ml), IL-12 (100 U/ml), IFN-γ (1000 U/ml) and ovalbumin (200 μg/ml) were also added. Th2 cells were cultured with IL-1 (1 ng/ml), IL-2 (50 U/ml), IL-4 (100 U/ml), IL-5 (100 U/ml), IFN-γ (1000 U/ml) conalbumin (100 μg/ml). Aminoguanidine (1 mM) was also added to the inhibit nitric oxide production in cultures where peritoneal macrophages were used as APC. The proliferation and cytokine secretion were estimated as described above.

### Statistics

Statistical analysis was done using unpaired student's t-test. '◆' Represents p < 0.05, '†' p < 0.01, '*' p < 0.001.

## Abbreviations

APC: antigen presenting cells; PEC: peritoneal exudates cells; PMQ: peritoneal macrophages; SMQ: splenic macrophages; IFN-γ: interferon-gamma, Ag: antigen.

## Authors' contributions

VS did all proliferation and cytokines assays, performed literature searches and drafted the manuscript. JNA conceived, coordinated and supervised the study, and prepared the manuscript. All authors read and approved the final manuscript.
